# Successive-cyclic movement in humans and neural language models: testing wh-filler-gap dependencies

**DOI:** 10.3389/fpsyg.2025.1699740

**Published:** 2026-01-08

**Authors:** Keonwoo Koo, Hyosik Kim

**Affiliations:** 1 Department of English Language and Literature, Dongguk University, Seoul, Republic of Korea; 2 Department of English Language and Literature, Jeonju University, Jeonju, Republic of Korea

**Keywords:** backward sluicing, intermediate structure, neural language models, poverty of stimulus, successive-cyclic movement, wh-filler-gap dependency

## Abstract

This study investigates whether auto-regressive language models (GPT-2, GPT-Neo, OPT) replicate human-like sensitivity to covert intermediate phrasal structures (CP vs. NP) during the processing of wh-filler-gap dependencies. We extend this inquiry to backward sluicing, an elliptical construction that provides a robust test for the representation of abstract syntactic structure. Across two experiments measuring processing difficulty via surprisal, we found a significant divergence from established human processing patterns. We found that the models failed to reproduce the human processing facilitation for both canonical and elided dependencies. One model, in fact, showed an inverse effect, a pattern suggesting a reliance on surface-level cues rather than abstract hierarchical representations. We take these findings as evidence that the tested GPT-style models are insufficient for deriving knowledge of covert syntactic structures. This failure lends empirical support to the Poverty of the Stimulus (PoS) argument, and also highlights a significant gap in the cognitive plausibility of contemporary NLMs as models of human syntactic competence.

## 1 Introduction

A central question in the psycholinguistics is how comprehenders incrementally construct and maintain long-distance dependencies in real time. In wh-filler-gap dependencies, a fronted wh-phrase must be linked to a later gap across potentially substantial syntactic distance. Numerous studies suggest that human parsing in such configurations is strongly structure-sensitive: comprehenders predict upcoming licensors and retrieve previously stored constituents on the basis of hierarchical representations rather than surface contiguity alone (Gibson and Warren, 2004; Keine, 2020; Kim et al., 2025).

The ability to comprehend and produce long-distance dependencies, such as wh-dependencies, is a hallmark of human syntactic competence. In generative syntax, successive cyclic movement has been proposed to account for the locality constraints governing such dependencies, positing that wh-elements move through intermediate syntactic positions—specifically the specifiers of CP—on their way to the final landing site (Abels, 2012; Chomsky, 1973; Chung et al., 1995; Henry, 1995; McCloskey, 1979, 2002; Takahashi, 1994; Torrego, 1984; Van Urk, 2020). Although these intermediate positions are not surface-visible, they are theoretically necessary to explain phenomena such as island effects and subjacency violations. For instance, consider the grammaticality contrast between (1) and (2):

(1) Who_i_ did the consultant claim *t*_i_ that the proposal had pleased *t*_i_?
(2) *Who_i_ did the consultant wonder which proposal had pleased *t*_i_? (Gibson and Warren, 2004, p. 56).

In (1), the embedded clause (CP) serves as an intermediate landing site for the wh-phrase *who*, allowing movement to proceed in two successive steps. In contrast, (2) contains another wh-phrase (*which proposal*) that occupies the specifier of the embedded CP, blocking the intermediate position. The wh-movement would cross two bounding nodes (TPs) at once, resulting in a subjacency violation (Chomsky, 1973).

This hypothesis, referred to as the Intermediate Structure Hypothesis by Gibson and Warren (2004), raises a foundational question in language acquisition: how can learners acquire abstract structural representations that are not directly observable in the linguistic input? This question lies at the heart of the Poverty of the Stimulus (PoS) argument, which posits that certain aspects of grammatical knowledge are not learnable from positive evidence alone, and therefore must be innately specified (Chomsky, 1981).

However, recent work in computational modeling has challenged the reach of the PoS argument. Wilcox et al. (2024), for example, demonstrate that Neural Language Models (NLMs) exhibit sensitivity to wh-movements and island constraints (Ross, 1967), even though such constraints are only rarely and indirectly evidenced in the training data. By analyzing surprisal values across grammatical and ungrammatical structures, their findings suggest that models can acquire abstract constraints from raw distributional input alone—relying on domain-general learning mechanisms (Clark and Lappin, 2010; Yang and Piantadosi, 2022), rather than requiring innate syntactic knowledge.

Building on this insight, the present study investigates whether NLMs also exhibit sensitivity to *successive cyclic movement*—a deeper structural principle that underlies island effects (e.g., wh-islands) but is less directly observable than the surface violations studied by Wilcox et al. Specifically, we ask whether these models represent or approximate the intermediate steps posited by generative grammar, and whether their processing of long-distance dependencies mirrors the incremental, structure-sensitive parsing seen in human comprehension. To preview our results, we failed to find evidence that NLMs integrate the intermediate structure during the processing of Wh-Filler-Gap Dependency (WhFGD), as humans do.

In exploring this question, we extend the investigation to ellipsis constructions, specifically backward sluicing, which provides a novel empirical window into the interaction between movement and ellipsis. Kim et al. (2025), for instance, show that the intermediate structure hypothesis confirmed in a canonical wh-dependency in human comprehension was also confirmed for backward sluicing, where wh-movement does not occur overtly, as shown in (3).

(3) I do not know which book [e], but John talked to Mary about a new book. (Kim et al., 2025).

Although there is no clear evidence that the wh-phrase *which book* undergoes movement—given that elements such as verbs, which could integrate with the wh-phrase, are absent in the ellipsis site—it was confirmed that the parser could not only interpret it as a correlate of *a new book* in the antecedent, thereby forming a WhFGD, but also sensitive to the intermediate structure of the antecedent. This suggests that humans can exploit intermediate structure at a deeper, more abstract level of representation.

Thus, in our study, in addition to canonical wh-questions, we analyze NLMs’ responses to backward sluicing constructions involving islands and potential intermediate CPs. By doing so, we aim to assess not only whether models can approximate surface constraints, but also whether they encode intermediate syntactic representations necessary for reconstructing ellipsis targets. If models succeed in replicating human-like behavior across both canonical and elliptical contexts, this would pose a significant challenge to the PoS argument. Conversely, systematic failures in such cases would highlight a continued gap between humans and NLMs.

This paper is organized as follows. In Section 2, we introduce wh-filler-gap dependencies and backward sluicing, and provide a brief overview of NLMs to capture wh-filler-gap dependencies. In Section 3, we outline the methodology used to evaluate NLMs’ intermediate structure when processing wh-filler-gap dependency and backward sluicing. We describe the three autoregressive GPT-style models tested, the surprisal-based metrics used to measure processing difficulty, and the test suites for the experiments. In Section 4, we present the results of two experiments designed to compare NLMs to humans on these questions. Experiment 1 focuses on wh-filler-gap processing and tests whether models are sensitive to the complexity of intermediate structures (CP vs. NP), while Experiment 2 extends these manipulations to backward sluicing and comparable non-elliptical wh-questions. In Section 5, we synthesize the experimental findings and discuss their implications for linguistic theory, regarding whether the absence or presence of intermediate structure effects in NLMs challenges or reinforces the PoS argument, and assess the extent to which NLMs can serve as cognitively plausible models of human syntactic processing.

## 2 The effect of intermediate structure on WhFGD processing of humans and NLMs

### 2.1 Wh-filler-gap dependency in human sentence processing

Wh-Filler-Gap Dependencies (WhFGDs) have been a key focus in linguistic theory for decades, as they involve complex syntactic mechanisms that extend beyond linear or surface patterns. This is because the filler and its associated gap can be separated by a long distance in the surface word order, yet still maintain a meaningful dependency (Chomsky, 1957; Ross, 1967). According to Chomsky (1973), long-distance dependencies crossing multiple clauses are mediated by an intermediate structure. Consistent with this, previous studies on the human processing of WhFGDs have shown that the structure between a *wh*-filler and its associated gap influences the formation of dependencies (Gibson and Warren, 2004; Keine, 2020; Kim et al., 2025). For instance, Gibson and Warren compared the processing of sentences in (4).

(4) a. The manager who_i_ the consultant claimed [_CP_ that the new proposal had pleased *t*_i_] will hire five workers tomorrow.
  b. The manager who_i_ [_NP_ the consultant’s claim about the new proposal] had pleased *t*_i_ will hire five workers tomorrow (Gibson and Warren, 2004).

In (1a), the gap associated with the *wh*-phrase is inside the embedded CP, while in (4b), the gap does not appear inside a WhFGD. In both cases, the structure of the elements after the verb *pleased* is changed to include either a CP or an NP. The hypothesis was that this structural difference would affect how long it takes to process the dependency, based on two main assumptions: (i) *wh*-phrases move through every CP position along their path, known as *Successive-Cyclic Movement*. (ii) The linear distance between the *wh*-phrase and its gap makes a difference in processing difficulty; shorter dependencies are easier to process.

**Figure 1:**
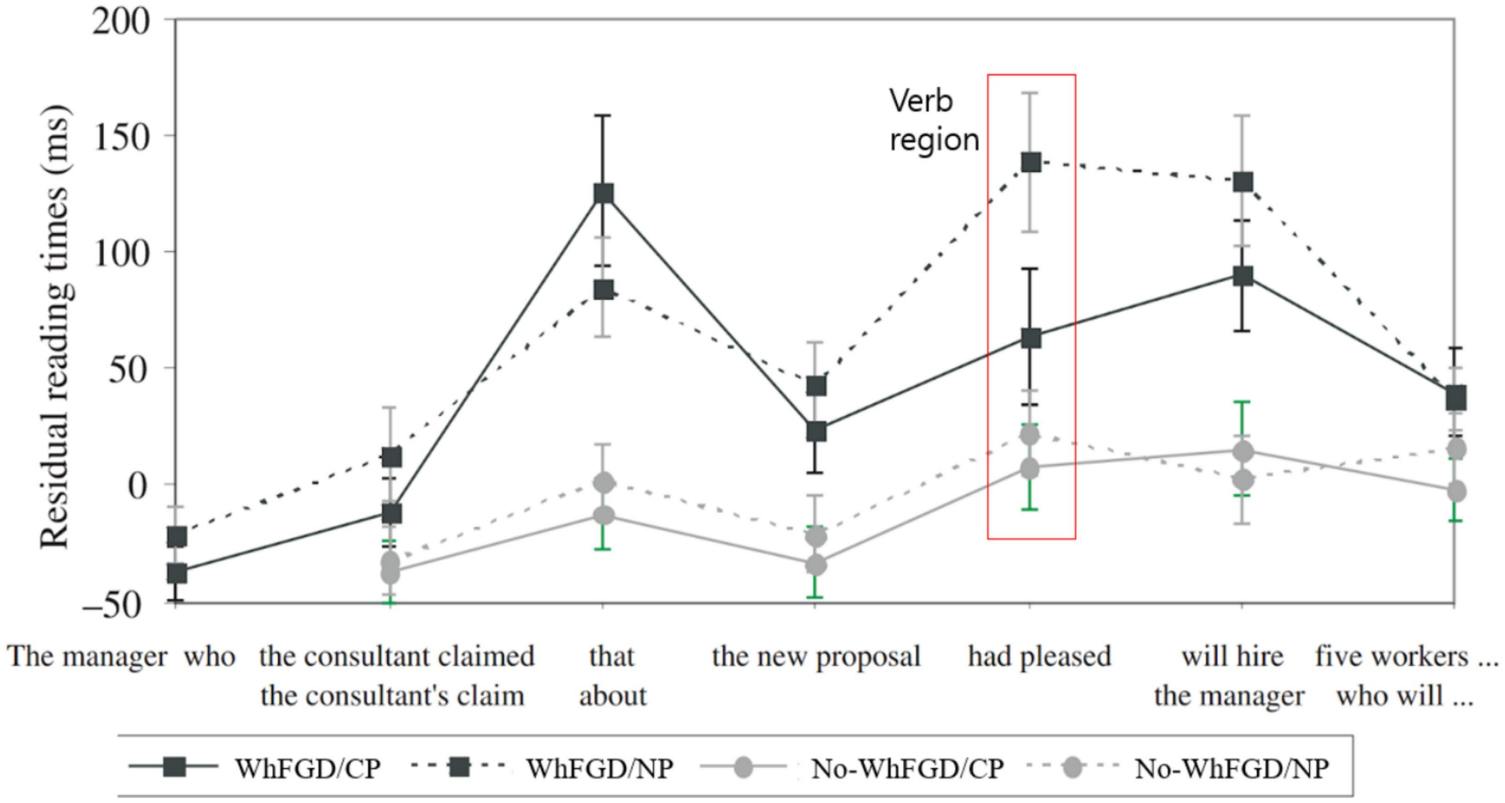
98 participants’ self-paced reading times for experiment 1. This figure is adapted from Gibson and Warren (2004).

Their results showed that processing the verb *pleased* was faster when a CP intervened in the dependency than when an NP did. Gibson and Warren argue that because of this cyclic movement of *wh*-phrases, the embedded CP in (4a) acts as an intermediate landing site for the *wh*-movement. This intermediate Spec-CP shortens the linear distance between the *wh*-phrase and the gap compared to (4b), where no embedded CP is present (see Figure 1 for their results). Thus, they attributed this result to the parser’s active use of knowledge about successive-cyclic movement.

Building on Gibson and Warren’s work, Kim et al. (2025) extended this line of inquiry to investigate whether humans’ sensitivity to intermediate structures persists in more abstract syntactic contexts, such as ellipsis. Kim et al. addressed this issue by examining Backward Sluicing (BwS), a form of ellipsis in which an elided clause [e] precedes its antecedent clause, as in (5).

(5) I do not know which book [e], but John talked to Mary about a new book.

(a) I do not know which book *John talked to Mary about*, but John talked to Mary about some book.
(b) ^#^I do not know which book *Mary bought*, but John talked to Mary about some book (Kim, 2023).^1^

In (5), the interpretation of the *wh*-phrase (*which manager*) in the first clause depends on retrieving the structure from the antecedent in the second clause. This resolution process is governed by a parallelism condition: the elided clause has to be structurally parallel to the antecedent, licensing interpretation (5a) while excluding (5b).

Given these properties of BwS, Kim et al. designed an experiment mirroring Gibson and Warren’s study. They manipulated the structure of the antecedent clause, contrasting an intermediate CP in (6a) with an NP in (6b).

(6) a. I do not know which manager_i_ [e], but the consultant claimed [_CP_ that the new proposal had pleased *t*_i_] one of the managers.

b. I do not know which manager_i_ [e], but [_NP_ the consultant’s claim about the new proposal had pleased *t*_i_ one of the managers (Kim et al., 2025).

In (6), both sentences illustrate instances of BwS, where a *wh*-phrase appears in the first conjunct without an overt clausal structure to license or interpret it. Despite this absence, the second conjunct following the discourse connective *but* provides the necessary clausal information to resolve the ellipsis. This allows the *wh*-phrase to be interpreted as part of a WhFGD, with the second clause supplying the antecedent. Importantly, this interpretive dependency spans over intermediate structures, either a CP or an NP, indicating that WhFGDs can form across these syntactic configurations. The fact that the interpretation of the ellipsis site is associated with the antecedent raises the possibility that the ellipsis site may contain a syntactic structure parallel to that of the antecedent (Lasnik, 2001; Merchant, 2001).

Indeed, Kim et al. found that processing was significantly easier in the CP condition than in the NP condition. This result suggests that the human actively constructs an abstract syntactic representation for the ellipsis site incrementally with an intermediate trace. The findings imply that the mechanisms governing long-distance dependencies, including successive-cyclic movement, operate irrespective of whether the syntactic structure is overtly realized.

Taken together, psycholinguistic studies reveal a core feature of the human’s syntactic processing: its reliance on intermediate structures extends beyond overt WhFGD processing, operating even when those structures exist at a more abstract level within elided constituents. This demonstration of abstract structural processing in humans, therefore, raises a compelling subsequent question: How do NLMs process intermediate structures in elliptical constructions, and do they fully replicate this successive-cyclic movement sensitivity of human behavior?

### 2.2 WhFGDs in NLMs

NLMs have been shown to encode shared syntactic representations across distinct constructions in structural priming (Prasad et al., 2019; Sinclair et al., 2022). Further studies demonstrate that NLMs can generalize to syntactic structures not explicitly represented in their training data, suggesting a capacity to abstract beyond surface-level patterns (Jumelet et al., 2021; Warstadt and Bowman, 2022; Misra and Mahowald, 2024). This has prompted researchers to examine whether NLMs approximate aspects of human syntactic learning, particularly in constructions that require hierarchical representations, such as subject–verb agreement and polar (yes/no) questions (Yedetore et al., 2023; Evanson et al., 2023).

One domain where this question has received focused attention is filler-gap dependencies. Recent findings indicate that NLMs exhibit sensitivity to such wh-dependencies and syntactic island constraints across languages (Wilcox et al., 2018; Bhattacharya and van Schijndel, 2020; Ozaki et al., 2022; Kobzeva et al., 2023; Wilcox et al., 2024). Notably, Wilcox et al. (2024) showed that NLMs can reliably distinguish between acceptable wh-movement structures and ungrammatical island violations, as in (7) and (8), in a manner that approximates human sensitivity. These results bear directly on long-standing debates in generative grammar, which have traditionally attributed such sensitivities to innate, domain-specific grammatical constraints (e.g., Chomsky, 1964).

(7) a. The fact that the reporter knows who [the witness surprised __ with his testimony] surprised the jury during the trial.
  b. *The fact that the reporter knows who [the witness shocked the jury with his testimony] surprised during the trial.

(8) a. I know who Alex said your friend insulted __ yesterday.
b. *I know who Alex said [_CP_ whether your friend insulted __ yesterday] (Wilcox et al., 2024).

The traditional *grammatical account*, rooted in the Poverty of the Stimulus argument, posits that sensitivity to island violations stems from innate linguistic constraints, as such violations are too rare in children’s input to be learned from experience alone (Chomsky, 1964). From this perspective, the fact that NLMs show human-like behavior challenges the PoS argument (Wilcox et al., 2024). If NLMs, which lack innate syntactic knowledge, can detect island violations, then these sensitivities could be learned from input, provided they possess the right inductive biases.

Supporting this alternative view, Phillips (2013) claims that island sensitivity can arise from language input, without needing innate syntactic knowledge. Since NLMs depend only on input-driven learning and inductive generalization, their success with phenomena such as wh-islands makes them useful for studying the learnability of syntax. In this context, wh-movement and island constraints serve as ideal test cases.

Given their nature as input-driven learners, NLMs provide a powerful tool for investigating the learnability of syntax. Their architecture, which starts with random initializations and uses large matrices to approximate a variety of functions, does not inherently limit their ability to capture complex linguistic generalizations (Wilcox et al., 2024). We therefore follow Clark and Lappin (2010) and Wilcox et al. (2024) in defining NLMs as domain-general learners. In this context, *wh*-movement, including successive-cyclic movement, serves as an ideal test case for exploring what can be learned from data alone.

Building on this, the current study examines how NLMs process *intermediate syntactic structures* in wh-movement and BwS—specifically, whether they show sensitivity to *successive-cyclic movement*. Although previous research shows that NLMs can identify island violation [e.g., *wh*-island in (8)], it remains an open question whether they also represent *intermediate landing sites* within these structures. Therefore, this study examines whether NLMs similarly benefit from intermediate structures when processing filler-gap dependencies and BwS, and whether their behavior aligns with or diverges from human syntactic processing strategies.

## 3 Methodology

### 3.1 Neural language models and metrics

We selected three GPT-style auto-regressive NLMs: GPT-2 (Radford et al., 2019), GPT-Neo (Black et al., 2022), and OPT (Zhang et al., 2022), trying to match their parameter sizes where possible.^2^ We chose auto-regressive models for their incremental, *left-to-right* processing, which aligns with previous research on this processing approach (Wilcox et al., 2018; Ozaki et al., 2022; Howitt et al., 2024; Lan et al., 2024; Wilcox et al., 2024; Boguraev et al., 2025). Although all three are auto-regressive, they represent distinct architectures and pretraining pipelines. In addition, each model has different implementation details and was trained on a distinct corpus (e.g., Webtext for GPT-2, The Pile for GPT-Neo, and OPT on a separately curated mixture of internet corpora). This choice allows us to test the generalizability of our findings (Hardware for compute environment is in Appendix A).

Building on prior research that employs Surprisal (Hale, 2001; Levy, 2008) as a metric for processing difficulty of NLMs (Smith and Levy, 2013; Goodkind and Bicknell, 2018; Wilcox et al., 2020; Shain et al., 2024; Ozaki et al., 2022; Howitt et al., 2024; Lan et al., 2024; Wilcox et al., 2024),^3^ we compute surprisal as: *S*_t_ = -log_2_*P*(*w_t_* | *w*_1_, *w*_2_, …., *w*_*t*-1_) where *w_t_* is the target word at position *t*, and the probability is conditioned on the preceding context *w*_1_, *w*_2_, …., *w*_*t*-1_. A lower probability corresponds to a higher surprisal: as the probability approaches zero, surprisal approaches infinity, whereas a probability approaching one yields a surprisal close to zero. In line with standard practice in computational psycholinguistics, we interpret surprisal as a linking hypothesis to human on-line processing measures such as self-paced reading times and eye-movements (Hale, 2001; Levy, 2008; Smith and Levy, 2013).

In addition, since the tested models are trained with a subword tokenizer (e.g., BPE), we adopt Oh et al. (2024)’s word-level surprisal method, negative log probabilities of subword tokens corresponding to *w_t_* were summed to calculate S(*w_t_*) = -log*P*(*w_t_* | *w*_1_…*w*_*t*-1_) according to the chain rule of conditional probabilities. Regarding the truncation policies of experimental sentences, the processing loop treated every sentence as a distinct sequence, which was explicitly initiated with the Beginning-of-Sentence (BOS) token. This guarantees that no contextual information or hidden state was carried over from preceding sentences during the calculation of surprisal. Overall, higher probability equates to lower surprisal; evidence in favor of a model’s syntactic knowledge is reflected in assigning lower surprisal to grammatical continuations than to ungrammatical ones—an effect that Wilcox et al. observed in *wh*-movement. Accordingly, we calculate surprisal incrementally, word-by-word, across all regions of each sentence, with a particular focus on the critical regions relevant to the dependency under investigation.

## 4 Experiments

Experiments 1 and 2 were designed to assess whether the NLMs under investigation genuinely encode the grammatical constraints underlying wh-movement.^4^ More specifically, we focus on the representation of intermediate structures, where humans exhibit sensitivity to the Spec-CP position—a hallmark of successive-cyclic movement. The structural complexity manipulations employed here draw on well-established configurations from prior research on WhFGD and backward sluicing (BwS) phenomena (Gibson and Warren, 2004; Keine, 2020; Kim et al., 2025). In Experiment 1, we applied manipulations previously validated in studies of WhFGD processing. Experiment 2 extended these manipulations to BwS constructions and structurally comparable non-elliptical wh-questions, thereby allowing us to determine whether the effects observed in Experiment 1 reflect structural complexity specifically associated with WhFGDs in elliptical contexts.

### 4.1 Experiment 1

Experiment 1 directly extends existing studies of NLMs to WhFGDs. We examine how WhFGD is processed with antecedents of varying structural complexity. Since we focus on intermediate structures, we compare the intermediate structure with CP versus NP. If NLMs process similarly to humans, we expect a surprisal gap between CP and NP conditions. We will refer to these effects as the Intermediate Structure Effects.

#### 4.1.1 Materials

The materials followed a 2×2 factorial design, where Intermediate Structure (CP vs. NP) and Structural Type (WhFGD vs. No-WhFGD) were manipulated as independent factors. A sample set of stimuli is presented in Table 1.

**Table 1:** Examples of experiment 1.

Condition	Examples of stimuli
WhFGD / CP	The manager who the consultant claimed that the new proposal had pleased will hire five workers tomorrow.
WhFGD / NP	The manager who the consultant’s claim about the new proposal had pleased will hire five workers tomorrow.
No-WhFGD / CP	The consultant claimed that the new proposal had pleased the manager who will hire five workers tomorrow.
No-WhFGD / NP	The consultant’s claim about the new proposal had pleased the manager who will hire five workers tomorrow.

The stimuli for this experiment comprised 24 items, drawn from Gibson and Warren (2004) and Keine (2020), and all items were carefully controlled for lexical and plausibility factors. As mentioned, past studies found that humans find it harder to process WhFGD where an intermediate NP follows a wh-filler before a gap (e.g., *wh-filler … NP … gap*) compared to when a CP follows a *wh*-filler before a gap (e.g., *wh-filler … CP … gap*; Gibson and Warren, 2004; Keine, 2020), as in Figure 1, which was adapted from Gibson and Warren.

In addition, as a reviewer pointed out that the CP and NP conditions are matched on local transitional probabilities that could differently affect surprisal independently of intermediate structure, we report the bigram transitional probabilities between the three-word pre-critical region and the critical region.

The analysis revealed identical transitional probabilities across syntactic conditions (M_CP = 0.108, M_NP = 0.109; *t* = −0.18, *p* = 0.85), indicating that CP and NP items do not differ in lexical predictability at the critical region. A similar pattern was observed for Structure Type (WhFGD vs. No-WhFGD: 0.104 vs. 0.112). Therefore, the surprisal reported in the main experiments cannot be attributed to local n-gram distribution and instead reflects the processing of hierarchical structure.

Thus, for NLMs, if intermediate structures influence the processing of WhFGD, we would expect to observe an Intermediate Structure effect in WhFGD/CP conditions. In this condition, the NLMs would attempt to integrate the *wh*-word with the verb *pleased* by reactivating the *wh*-word. Due to this integration, the distance between the *wh*-word and the verb *pleased* would be shorter when the CP-structure is involved compared to an intermediate NP-structure.

#### 4.1.2 Data analysis and results

**Figure 2:**
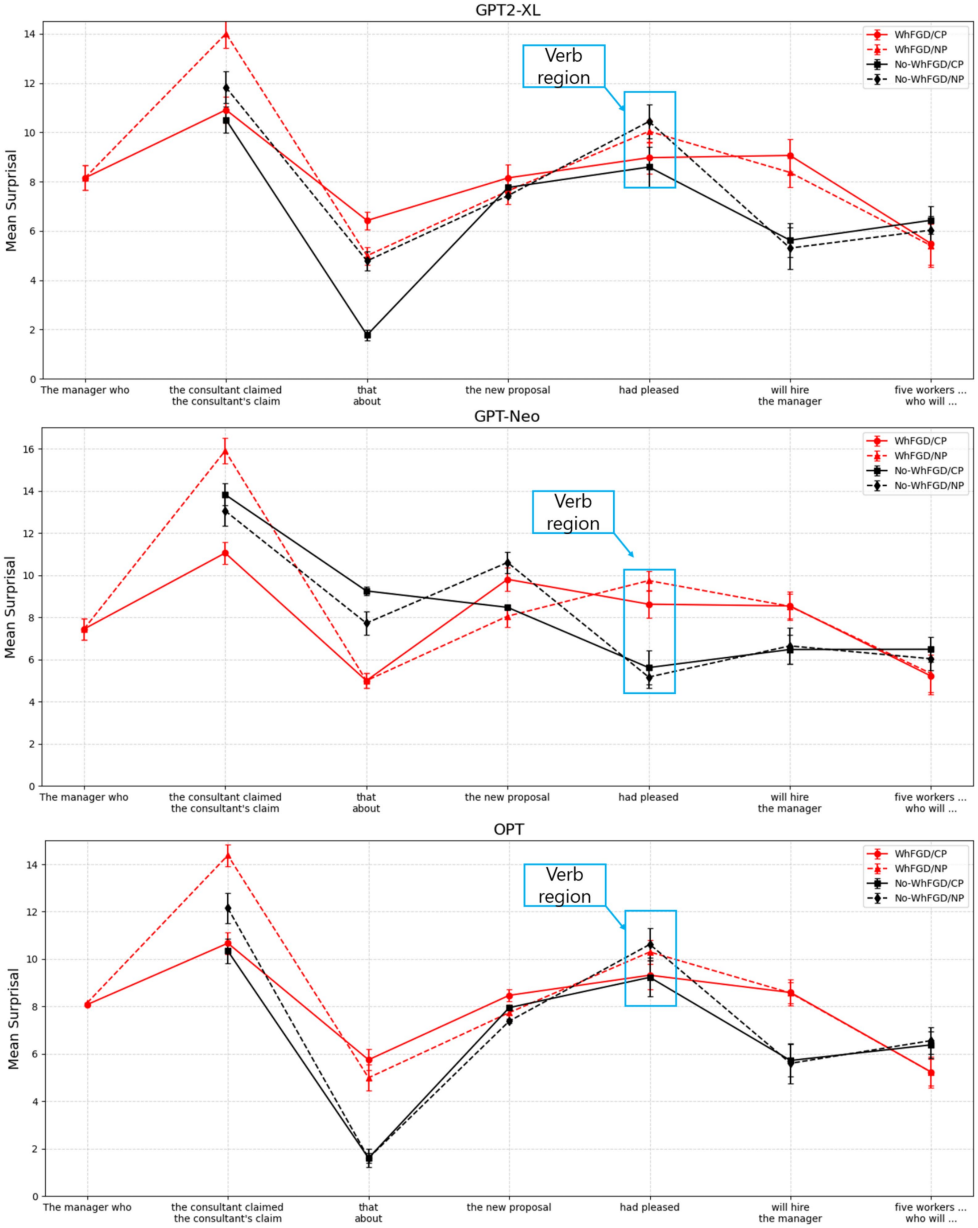
Mean surprisal for each region in WhFGD and No-WhFGD conditions of NLMs. Error bars represent 95% confidence intervals.

**Figure 3:**
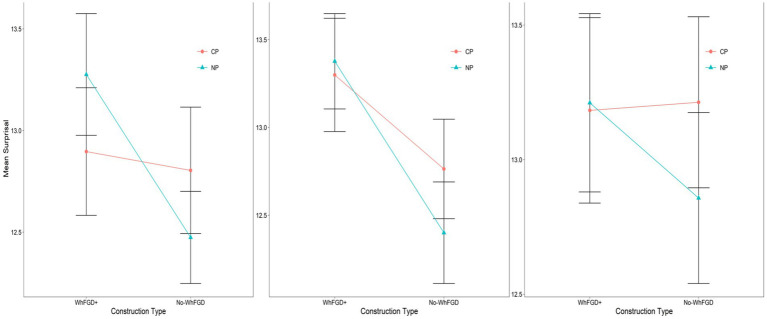
Mean surprisal for critical region (“pleased”) in WhFGD and No-WhFGD conditions (GPT2-XL: Left, GPT-Neo: Mid, OPT: Right). Error bars represent 95% confidence intervals.

Data analysis was conducted using R software (Team, R. C, 2021). A linear mixed-effects model was employed to analyze the surprisal for each region. The analysis was performed using the *lmer* function from the *lme4* package (Bates et al., 2014), with Intermediate Structure and Construction Type as fixed effects, incorporating a maximally convergent random effects structure (Barr et al., 2013) that included by-item random intercepts and random slopes for both Construction Type and Intermediate Structure. Fixed effects were sum-contrast coded (Construction Type: WhFGD = −0.5, No-WhFGD = 0.5; Intermediate Structure: CP = −0.5, NP = 0.5).^5^ All *p*-values were calculated using the *lmerTest* package (Kuznetsova et al., 2017). To directly compare the results with those of humans in Gibson and Warren’s study, we grouped the regions and calculated the mean surprisal. We report both the all-region plot in Figure 2 and the critical region in Figure 3.

For GPT2-XL, at the critical region “*pleased,”* a linear mixed-effects model revealed no significant main effect of Intermediate Structure (*β* = 0.02, *SE* = 0.47, *t* = 0.05, *p* > 0.1). Also, no significant main effect of Construction Type was detected (*β* = −0.45, *SE* = 0.35, *t* = −1.28, *p* > 0.1), nor was there an interaction effect (*β* = −0.7, *SE* = 0.43, *t* = −1.64, *p* > 0.1).

In the case of GPT-Neo, a linear mixed-effects model showed no significant main effect of Intermediate Structure (*β* = −0.14, *SE* = 0.49, *t* = −0.3, *p* > 0.1). However, a marginal main effect of Construction Type was observed (*β* = −0.76, *SE* = 0.40, *t* = −1.91, *p* = 0.068), suggesting a trend toward higher surprisal in the WhFGD condition compared to the No-WhFGD condition. The interaction was not significant (*β* = −0.44, *SE* = 0.56, *t* = −0.78, *p* > 0.1). This indicates that the model was responsive to construction type (WhFGD vs. No-WhFGD), but not to intermediate structure (CP vs. NP). Critically, effect-size analyses based on pairwise comparisons showed that the marginal main effect of Construction Type did not translate into a measurable Intermediate Structure effect. For pairwise contrasts, we additionally report *Δ*surprisal (in bits) with 95% confidence intervals as an effect size. The CP–NP difference in surprisal was negligible in both construction types: Δ = −0.08 bits, 95% CI [−1.21, 1.06] in the Wh-FGD condition and Δ = 0.36 bits, 95% CI [−0.77, 1.50] in the No-WhFGD condition (both *p*s> 0.1). Thus, although GPT-Neo exhibited a weak sensitivity to Construction Type, it did not encode the intermediate landing site that characterizes human processing of successive-cyclic movement.

Regarding the OPT model, Intermediate Structure (*β* = −0.16, *SE* = 0.64, *t* = −0.25, *p* > 0.1), Construction Type (*β* = −0.16, *SE* = 0.39, *t* = −0.41, *p* > 0.1), and interaction effect (*β* = −0.38, *SE* = 0.58, *t* = −0.65, *p* > 0.1) were not significant; thus, it did not show any main effects. The summary of results is in Table 2.

**Table 2:** Result summary of experiment 1.

Models	β intermediate structure (CP vs. NP)	β construction type	β interaction	Δ surprisal (bits)	95% CI	*p*
GPT2-XL	0.02 (*SE* = 0.47, *t* = −0.05, *p* > 0.1)	−0.45 (*SE* = 0.35, *t* = −1.28, *p* > 0.1)	−0.71 (*SE* = 0.43, *t* = −1.64, *p > 0*.1)	≈ 0 bits	CI spans zero	ns
GPT-Neo	−0.14 (*SE* = 0.49, *t* = −0.29, *p* > 0.1)	−0.76 (*SE* = 0.40, *t* = −1.91, *p* = 0.068)	−0.44 (*SE* = 0.56, *t* = −0.79, *p > 0*.1)	≈ 0 bits	CI spans zero	ns
OPT	−0.16 (*SE* = 0.65, *t* = −0.25, *p* > 0.1)	−0.16 (*SE* = 0.40, *t* = −0.41, *p* > 0.1)	−0.38 (*SE* = 0.58, *t* = −0.66, *p > 0*.1)	≈ 0 bits	CI spans zero	ns

To sum up, the GPT2-XL and OPT models did not show any significant main effect, and only the GPT-Neo showed a marginal construction type effect. Therefore, the results indicate that all three models are not sensitive to successive-cyclic movement, which is an intermediate structure.

#### 4.1.3 Discussion

In the WhFGD conditions, none of the three NLMs exhibited the Intermediate Structure effects reported in human sentence processing studies, those by Gibson and Warren (2004). In human processing, increased syntactic complexity between a *wh*-filler and its associated gap typically incurs higher processing costs, particularly when the intermediate structure is an NP rather than a CP, since CPs serve as intermediate landing sites (Spec-CP). Previous studies have demonstrated that NP-intervening structures delay processing at the critical verb region (e.g., “*pleased*”), suggesting that humans are sensitive to the intermediate syntactic structures of wh-filler-gap dependencies (see humans’ result in Figure 1).

In contrast, the NLMs analyzed here showed no significant increase in surprisal for the WhFGD/NP condition, indicating they are insensitive to the syntactic distinction between intermediate NP and CP. This lack of effect suggests that NLMs fail to exploit the hierarchical properties associated with intermediate wh-movement. The absence of such facilitation effects in the NLMs implies that they do not represent or integrate successive-cyclic movements regarding wh-movement in a human-like manner.

However, while GPT2-XL and OPT failed to even differentiate between WhFGD and No-WhFGD constructions, GPT-Neo exhibited a significant distinction between the two. One could posit that GPT-Neo acquired the ability to distinguish construction types; however, consistent with the other models, it did not demonstrate evidence of sensitivity to intermediate structural complexity. This suggests that although GPT-Neo may have learned to distinguish construction types, it cannot still model intermediate syntactic representations in a manner comparable to human processing.

### 4.2 Experiment 2

Backward sluicing provides a specific test of the human comprehension system’s ability to reconstruct abstract structures in real-time. In these configurations, an apparently bare wh-phrase in the first conjunct must be interpreted against an elided clausal structure that follows later in the sentence. Therefore, successful interpretation requires the parser to (i) retrieve an appropriate antecedent clause, (ii) reconstruct a clausal structure parallel to the ellipsis site, and (iii) establish a wh-filler–gap dependency within that reconstructed structure (Kim et al., 2025). If intermediate representations such as Spec-CP landing sites are deployed even when no overt movement occurs, then CP-over-NP facilitation at the embedded verb should re-emerge in backward sluicing, just as it does in canonical wh-questions. Experiment 2 leverages this logic to ask whether the tested NLMs surprisal response during ellipsis resolution reflect the same abstract, incremental reconstruction structure.

#### 4.2.1 Materials

The materials followed a 2 × 2 factorial design, where Intermediate Structure (CP vs. NP) and Construction Type (Backward Sluicing: BwS vs. Wh-Question: Wh-Q) were manipulated as independent factors. A sample set of stimuli is presented in Table 3, and the stimuli for this experiment comprised 24 items. We adopted the stimuli from Kim et al. (2025).

**Table 3:** Examples of experiment 2.

Condition	Examples of stimuli
(a) BwS / CP	I do not know which manager, but the consultant claimed that the new proposal had pleased and satisfied one of the managers.
(b) BwS / NP	I do not know which manager, but the consultant’s claim about the new proposal had pleased and satisfied one of the managers.
(c) Wh-Q / CP	I do not know which manager the consultant claimed that the new proposal had pleased and satisfied.
(d) Wh-Q / NP	I do not know which manager the consultant’s claim that the new proposal had pleased and satisfied.

**Figure 4:**
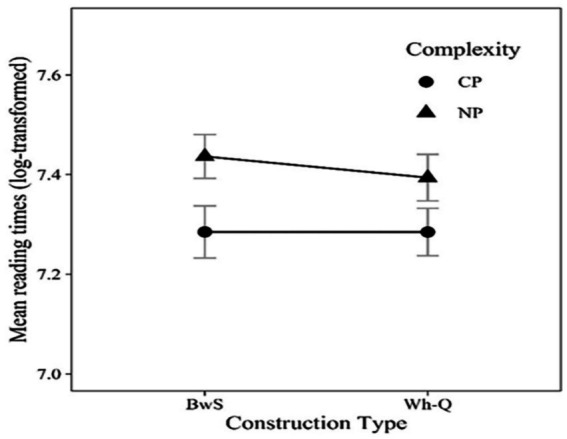
120 participants’ mean reading times for the critical region “*pleased*” in Experiment 2. This figure is adapted from Kim et al. (2025). Since this figure only shows the critical region, see the all-region plot in Kim et al. (2025).

In these conditions, if processing of WhFGDs is influenced by intermediate syntactic structures as humans (Gibson and Warren, 2004; Keine, 2020; Kim et al., 2025), we anticipate observing an Intermediate Structure effect in the Wh-Q conditions. Specifically, in the Wh-Q/CP condition, the parser is expected to facilitate integration between the *wh*-word and the verb “*claim*” by reactivating the *wh*-word at the intermediate CP position. This reactivation effectively shortens the syntactic distance between the *wh*-word and the embedded verb “*pleased*,” compared to cases where an intermediate NP structure is present. A similar prediction applies to BwS conditions, provided that the ellipsis site’s syntactic structure parallels that of its antecedent. Human result for the critical region (“*pleased*”) is illustrated in Figure 4, which is adapted from Kim et al.

#### 4.2.2 Data analysis and results

**Figure 5:**
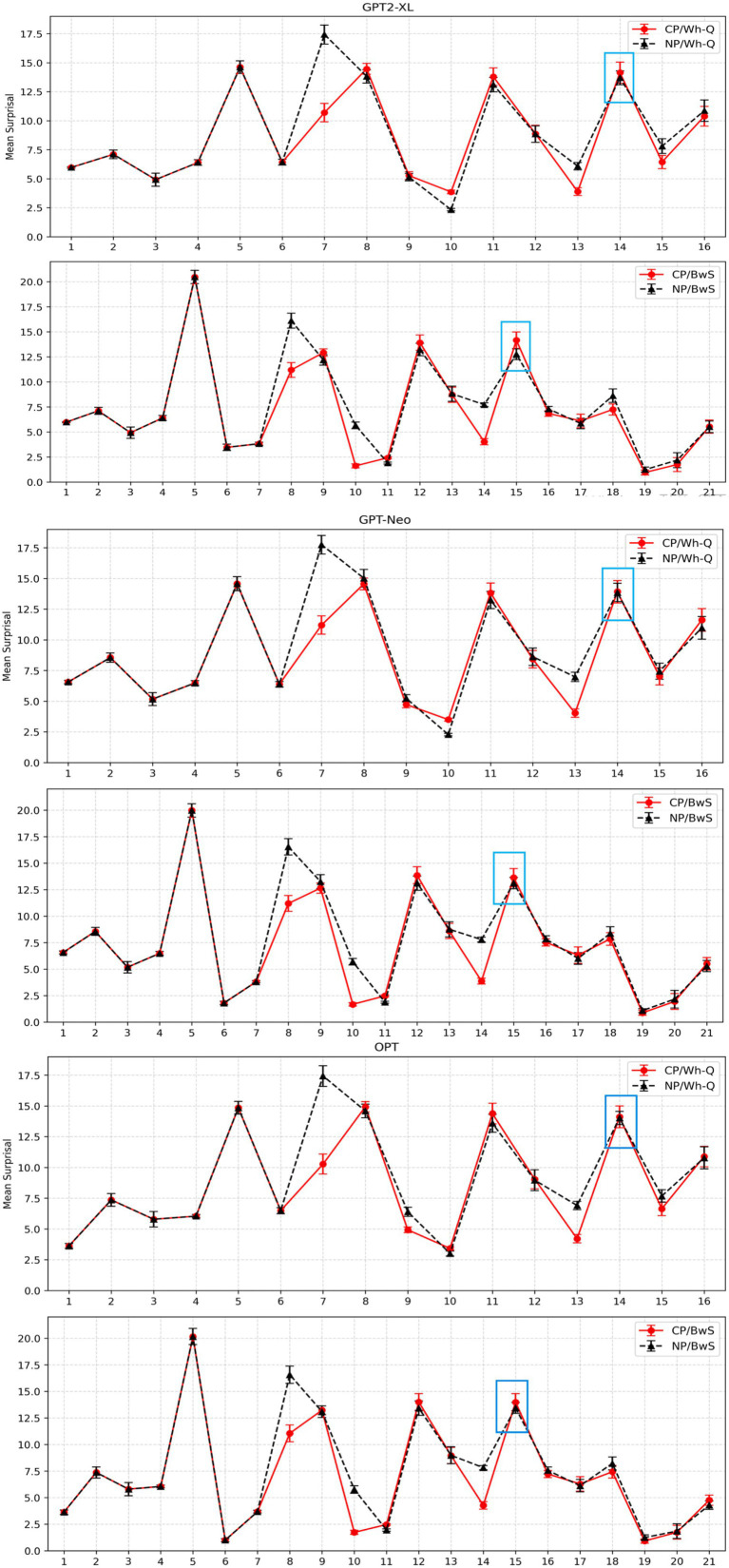
Three models’ mean surprisal for each region in Wh-Q and BwS conditions. Error bars represent 95% confidence intervals.

**Figure 6:**
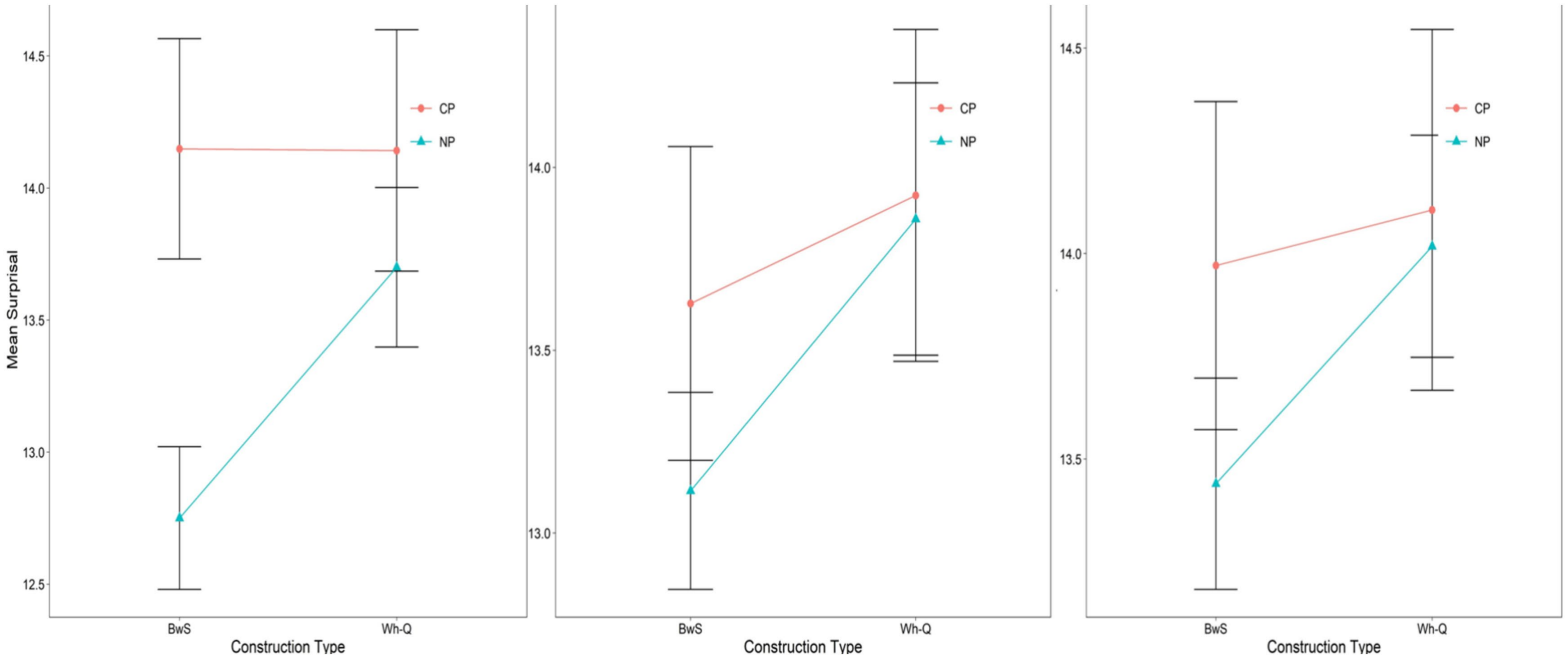
NLMs mean surprisal for critical region (“pleased”) in Wh-Q and BwS conditions (GPT2-XL: Left, GPT-Neo: Mid, OPT: Right). Error bars represent 95% confidence intervals.

A linear mixed-effects model was employed to analyze the surprisal for each region. The analysis was conducted using the *lmer* function from the *lme4* package (Bates et al. 2014), with intermediate structure and construction type as fixed effects, including by-item random intercepts and random slopes for both Construction Type and Intermediate Structure. Fixed effects were sum-contrast coded (Construction Type: BwS = −0.5, Wh-Q = 0.5; Intermediate Structure: CP = -0.5, NP = 0.5).^6^ All *p*-values were calculated using the *lmerTest* package. Figure 5 shows an all-region plot in the BwS and Wh-Q conditions with the critical region in a blue box. The specified critical region (“*pleased*”) plot in both constructions is depicted in Figure 6.

For GPT2-XL, a linear mixed effects model revealed no significant main effect of Intermediate Structure (*β* = −0.92, *SE* = 0.63, *t* = −1.45, *p* > 0.1), and no significant main effect of Construction Type (*β* = −0.47, *SE* = 0.45, *t* = −1.0, *p* > 0.1). However, a marginal interaction effect emerged (*β* = −0.96, *SE* = 0.50, *t* = −1.9, *p* = 0.069), suggesting that the CP-NP contrast might depend on construction type. Pairwise comparisons using the *lsmeans* package (Lenth, 2016) further clarified this pattern. For pairwise contrasts, we additionally report *Δ*surprisal (in bits) with 95% confidence intervals as an effect size. In the BwS condition, surprisal was significantly higher for CP than NP (Δ = 1.40 bits, *SE* = 0.68, 95% CI [0.01, 2.79], *p* = 0.049), whereas no CP-NP difference was observed in the Wh-Q condition (Δ = 0.44 bits, *SE* = 0.68, 95% CI [−0.95, 1.83], *p* = 0.52). Thus, the marginal interaction was driven entirely by a robust CP-over-NP surprisal difference in Backward Sluicing, indicating that GPT2-XL shows Intermediate-Structure sensitivity selectively in BwS configurations, still an *opposite* trend compared to humans.

In the case of the GPT-NEO, a linear mixed effects model revealed no significant main effect of Intermediate Structure (*β* = −0.29, *SE* = 0.64, *t* = −0.45, *p* > 0.1). Also, no significant main effect of Construction Type was detected (*β* = 0.52, *SE* = 0.47, *t* = 1.1, *p* > 0.1). In addition, the GPT-NEO did not show a significant interaction effect (*β* = 0.44, *SE* = 0.50, *t* = 0.88, *p* > 0.1).

Regarding the OPT model, same as GPT-NEO, Intermediate Structure (*β* = −0.31, *SE* = 0.65, *t* = −0.47, *p* > 0.1), Construction Type (*β* = 0.35, *SE* = 0.38, *t* = 0.93, *p* > 0.1), and interaction effect (*β* = 0.44, *SE* = 0.54, *t* = 0.81, *p* > 0.1) were not significant. Table 4 shows the summary of the overall results.

**Table 4:** Result summary of experiment 2.

Models	β intermediate structure (CP vs. NP)	β construction type	β interaction	Δ surprisal (bits)	95% CI	*p*
GPT2-XL	−0.92 (*SE* = 0.63, *t* = −1.45, *p* > 0.1)	−0.47 (*SE* = 0.45, *t* = −1.45, *p* > 0.1)	−0.96 (*SE* = 0.50, *t* = −1.90, *p = 0*.069)	1.40 bits	[0.01, 2.79]	Signf.
GPT-Neo	−0.29 (*SE* = 0.64, *t* = −0.45, *p* > 0.1)	0.52 (*SE* = 0.47, *t* = −1.10, *p* > 0.1)	0.45 (*SE* = 0.51, *t* = 0.89, *p > 0*.1)	≈ 0 bits	CI spans zero	*ns*
OPT	−0.31 (*SE* = 0.66, *t* = −0.47, *p* > 0.1)	0.36 (*SE* = 0.38, *t* = 0.93, *p* > 0.1)	0.44 (*SE* = 0.54, *t* = 0.82, *p > 0*.1)	≈ 0 bits	CI spans zero	*ns*

#### 4.2.3 Discussion

Results from the GPT-Neo and OPT models indicate that neither model shows sensitivity to the Intermediate Structure effect in the Wh-Question or Backward Sluicing conditions, suggesting their behavior differs from human processing. Furthermore, GPT2-XL did demonstrate a trend of sensitivity to structural differences in the BwS condition, but the direction of this effect was *opposite* to what is seen in humans. While human participants process significantly faster in intermediate CP-structures than in NP-structures, as in Figure 4 (Kim et al., 2025), GPT2-XL showed lower integration costs for NP-structures when linking the *wh*-word to the verb within the ellipsis site. Kim et al. (2025) attribute the human pattern to the facilitating role of an intermediate Spec-CP position in CP structures, which eases processing difficulty. Conversely, GPT2-XL showed a trend of higher surprisal to CP structures than NP structures in the BwS condition, indicating that its behavior seems to rely on surface-level statistical patterns rather than syntactic representations involving an intermediate Spec-CP position.

Additionally, the lack of an Intermediate Structure effect in Wh-Question conditions suggests that all models’ processing of wh-filler-gap dependencies is not affected by the syntactic configuration of the dependency itself. In human processing, Kim et al. report clear complexity effects: NP-structures require more processing effort than CP structures because CP-structures provide an intermediate Spec-CP position that acts as a landing site for the *wh*-word, reducing the syntactic distance between the *wh*-word and the gap. In NP-structures, the absence of such an intermediate position increases cognitive demands and results in slower reading times (see humans’ result in Figure 4). All models’ failure to show this Intermediate Structure effect highlights a fundamental difference in parsing strategies between the NLMs and human language comprehension.

## 5 General discussion

Across two experiments, we find a fundamental divergence between humans and NLM sentence processing with respect to abstract syntactic representations. While humans incrementally project and use intermediate structures during the processing of wh-filler–gap dependencies; the NLMs we tested did not. In both long-distance wh-dependencies and backward sluicing, humans show clear sensitivity to the successive-cyclic movement that posits intermediate landing sites, whereas the models fail to capture these unobservable representations. Apparent NLM’s success on some constraints (e.g., certain island effects) therefore likely reflects learning of shallow, surface-level distributional patterns rather than acquisition of deep, hierarchical structure.

### 5.1 The implications of the PoS

With respect to the PoS debate, our tested GPT-style models’ results provide evidence that linguistic input alone and domain-general learning are insufficient for acquiring a human-like grammar. Intermediate syntactic positions, such as the Spec-CP landing sites in wh-movement, are not present in the surface input, making them a crucial test case. While Wilcox et al. (2024) have shown NLM sensitivity to island violations, our findings align with Lan et al. (2024), demonstrating that this success is superficial: When tested on phenomena that rely on deeper structural knowledge, NLMs fail. Consider (8), repeated below.

(8) a. I know who Alex said your friend insulted __ yesterday.
b. *I know who Alex said [_CP_ whether your friend insulted __ yesterday] (Wilcox et al., 2024).

Higher probability for (8a) over (8b) indicates some sensitivity, but this must not need reflect a representation of hierarchical dependencies; models may instead rely on cues such as the presence of complementizers (e.g., *whether*, *if*, etc.) crucially, in our paradigm NLMs did not respond in a human-like way to contrasts that hinge on intermediate structure (CP vs. NP). This failure to track successive-cyclic movement, even after exposure to massive corpora. The result strengthens the classic PoS argument: humans appear to be born with certain innate linguistic capacities, whereas our models are not.

This distinction is crucial. Intermediate syntactic positions, such as the Spec-CP that acts as a landing site in wh-movement, are not observed in the input and therefore serve as a key test for the PoS argument. While surface violations of island constraints may be learnable via distributional patterns, the processing of intermediate structures requires an understanding of hierarchical dependencies that are not explicitly marked in the data.

Thus, the failure of these models to capture such a core component of human sentence processing reinforces the challenges laid out in the PoS argument. Wilcox et al. (2024) argue that the success of NLMs on a range of wh-movement and island violations provides empirical evidence against the PoS for those structures. They suggest that domain-general learning algorithms, given sufficient data, can acquire these complex constraints without innate, language-specific biases. However, as Lan et al. (2024) counter, the success of NLMs is often limited to simpler constructions and breaks down when tested on more complex but related phenomena, such as parasitic gaps and across-the-board movement. Their work shows that even models trained on massive datasets fail to approximate human knowledge in these contexts. Our findings align with this latter view: the specific insensitivity to *intermediate* structure points to a continued gap between model performance and human competence that cannot be easily dismissed.

### 5.2 NLMs as cognitive models

These findings bear important implications for the application of NLMs as cognitive models. While Piantadosi (2023) posited that current NLMs offer a superior explanation of linguistic cognition compared to generative theories, Ziv et al. (2025) suggest viewing LLMs not as direct theories of human linguistic cognition, but as “proxies” for linguistically-neutral theories. Under this “Proxy View,” the NLMs function not as direct theories of human linguistic cognition, for which they are insufficient, but rather as proxies for linguistically-neutral theories, thereby reflecting the potential achievements of a capable learner. Our results challenge the stronger cognitive-model claim: if LLMs, when regarded as human cognitive models, consistently fail to acquire knowledge of intermediate structures despite successfully handling surface island constraints, it indicates that linguistically-neutral learners would also fail. This reinforces the idea that humans are *not* linguistically neutral and possess innate linguistic knowledge, structure-dependent processing.

This conclusion has direct ramifications for long-standing theories of syntactic learning. First, given that NLMs do not replicate human sensitivity to these intermediate structures, arguing for a reconsideration of the PoS argument based on successes with grammaticality tasks seems premature. Second, this divergence fundamentally questions the viability of using these NLMs as cognitive models for human linguistic knowledge (e.g., Piantadosi, 2023). A plausible model must not only predict grammaticality but also emulate the incremental mechanisms and processing characteristics of human comprehension; these models do not yet meet.

## 6 Conclusion

The current study evaluated whether contemporary neural language models display human-like sensitivity to intermediate syntactic representations in both wh-filler–gap dependencies and backward sluicing. Comparing predictions from three autoregressive models with established humans, we found that the models failed to reproduce the CP-over-NP facilitation that characterizes human sensitivity to intermediate landing sites; in backward sluicing, where humans incrementally reconstruct the elliptical structure, the models again showed reversed or even absent sensitivity. These patterns point to reliance on shallow distributional cues rather than hierarchical, incrementally deployed representations and therefore do not, on their own, weaken the Poverty of the Stimulus argument. While we stop short of claiming innateness as the only explanation, our results suggest that tested input-driven learning in diverse GPT-style architectures is insufficient to yield human-like, deeper structure representations. Future work could probe alternative inductive biases and training regimes, child-directed corpora, or larger models to test whether such sensitivities can emerge under different learning conditions.

## 7 Limitation

We also acknowledge several additional limitations of the present work. First, we restricted our investigation to English wh-filler–gap dependencies and backward sluicing, so experiments in other languages might yield different patterns. Second, we tested only relatively small GPT-style autoregressive models, and thus our negative findings cannot be straightforwardly generalized to all architectures or to larger LLMs (e.g., Llama-3, Pythia); future research should therefore examine a broader range of models. Finally, as noted by Kuribayashi et al. (2025), comparing surprisal across *internal layers* of NLMs may provide further insight into how model behavior relates to human sentence processing.

## Data Availability

The datasets presented in this study can be found in online repositories. The names of the repository/repositories and accession number(s) can be found in the article/supplementary material.

## Footnotes

1 (5b) can be a possible interpretation with approate contexts, but not in (5).

2 Specifically, GPT2-XL: (1.5B; https://huggingface.co/openai-community/gpt2-xl), GPT-Neo: (1.3B; https://huggingface.co/EleutherAI/gpt-neo-1.3B), and OPT: (1.3B; https://huggingface.co/facebook/opt-1.3b). Replications for checkpoints and tokenizers used are available on the above.

3 However, Huang et al. (2024) present arguments suggesting that surprisal does not consistently provide an accurate reflection of human behavior, particularly in the case of ambiguous sentences.

4 Data, analysis code, and experimental materials are available at: https://osf.io/gpc9h/overview?view_only=9d743bce1e184448ae48a690c81c54dc

5 model = lmer(surprisal ~ Construction * Intermediate Structure) + (Intermediate Structure + Construction | item).

6 model = lmer(surprisal ~ Construction * Intermediate Structure) + (Intermediate Structure + Construction | item).

## Appendix A

Hardware for experiment execution.

**Table:**

Resource type	Description
GPU	NVIDIA GeForce RTX 5000×4
CPU	I9-10980XE
RAM	32 GB
Evaluation Run	4 GPUs with 16GiB RAM
